# Replication of *Brucella abortus* and *Brucella melitensis* in fibroblasts does not require Atg5-dependent macroautophagy

**DOI:** 10.1186/s12866-014-0223-5

**Published:** 2014-09-02

**Authors:** Isabelle Hamer, Emeline Goffin, Xavier De Bolle, Jean-Jacques Letesson, Michel Jadot

**Affiliations:** Research Unit in Molecular Physiology (URPhyM), NAmur Research Institute for LIfe Sciences (NARILIS), University of Namur, Namur, Belgium; Research Unit in Biology of Microorganisms (URBM), NAmur Research Institute for LIfe Sciences (NARILIS), University of Namur, Namur, Belgium; Present address: Faculty of Veterinary Medicine-Department of infectious and parasitic diseases, Laboratory of Immunology and Vaccinology, University of Liège, Liège, Belgium

**Keywords:** *Brucella abortus*, *Brucella melitensis*, Intracellular trafficking, Replication, Macroautophagy, Atg5

## Abstract

**Background:**

Several intracellular bacterial pathogens have evolved subtle strategies to subvert vesicular trafficking pathways of their host cells to avoid killing and to replicate inside the cells. *Brucellae* are Gram-negative facultative intracellular bacteria that are responsible for brucellosis, a worldwide extended chronic zoonosis. Following invasion, *Brucella abortus* is found in a vacuole that interacts first with various endosomal compartments and then with endoplasmic reticulum sub-compartments. *Brucella* establishes its replication niche in ER-derived vesicles. In the past, it has been proposed that *B. abortus* passed through the macroautophagy pathway before reaching its niche of replication. However, recent experiments provided evidence that the classical macroautophagy pathway was not involved in the intracellular trafficking and the replication of *B. abortus* in bone marrow-derived macrophages and in HeLa cells. In contrast, another study showed that macroautophagy favoured the survival and the replication of *Brucella melitensis* in infected RAW264.7 macrophages. This raises the possibility that *B. abortus* and *B. melitensis* followed different intracellular pathways before replicating. In the present work, we have addressed this issue by comparing the replication rate of *B. abortus* and *B. melitensis* in embryonic fibroblasts derived from wild-type and Atg5^−/−^ mice, Atg5 being a core component of the canonical macroautophagic pathway.

**Results:**

Our results indicate that both *B. abortus* S2308 and *B. melitensis* 16M strains are able to invade and replicate in Atg5-deficient fibroblasts, suggesting that the canonical Atg5-dependent macroautophagic pathway is dispensable for *Brucella* replication. The number of viable bacteria was even slightly higher in Atg5^−/−^ fibroblasts than in wild-type fibroblasts. This increase could be due to a more efficient uptake or to a better survival rate of bacteria before the beginning of the replication in Atg5-deficient cells as compared to wild-type cells. Moreover, our data show that the infection with *B. abortus* or with *B. melitensis* does not stimulate neither the conversion of LC3-I to LC3-II nor the membrane recruitment of LC3 onto the BCV.

**Conclusion:**

Our study suggests that like *Brucella abortus, Brucella melitensis* does not subvert the canonical macroautophagy to reach its replicative niche or to stimulate its replication.

**Electronic supplementary material:**

The online version of this article (doi:10.1186/s12866-014-0223-5) contains supplementary material, which is available to authorized users.

## Background

Many intracellular bacteria have developed strategies to hijack the intracellular trafficking machinery of the host cell in order to escape lysosomal degradation ensuring their survival and their replication [1]. For example, *Mycobacterium tuberculosis* blocks the maturation of phagosomes into the degradative phagolysosomes by producing lipids that mimic the phosphoinositides and inhibit the fusion between phagosomes and lysosomes [2]. Some bacteria, including *Coxiella burnetii, Legionella pneumophila* and *Staphylococcus aureus* can survive and replicate for some time in autophagosome-like vacuoles by delaying [3,4] or by blocking [5] their maturation into autophagolysosomes.

After its uptake by HeLa cells, *Brucella abortus* is recovered in a vacuole (BCV) that transiently interacts with early and late endosomes and perhaps lysosomes, successively acquiring markers of endosomal compartments such as EEA1 (Early Endosome Antigen 1), Rab5, Rab7 and LAMP-1 (Lysosomal-associated membrane protein 1) [6]. During these different steps of maturation, the BCV becomes acidic allowing the expression of genes encoding the VirB type IV secretion system (T4SS) [6]. *Brucella* avoids lysosomal degradation by blocking the phagosome-lysosome fusion probably by a mechanism dependent on lipid rafts and perhaps on cyclic ß-1,2-glucans [7–9]. Afterwards, the BCV interacts in a sustained way with subdomains of the endoplasmic reticulum, called ERES (endoplasmic reticulum exit sites) and at around 12 h p.i., *Brucella abortus* starts to replicate in ER-derived vesicles labelled with ER specific markers, such as sec61ß and calnexin [6,10,11]. Later on, from 48 h p.i., Starr et al. [12] demonstrated that these replicative BCV (rBCV) could be converted into LAMP-1 and Rab7-positive compartments (called autophagic BCV or aBCV) that would be involved in the completion of the intracellular *Brucella* lifecycle and could promote its cell-to-cell spreading [12].

Earlier studies had already revealed that some bacteria resided in autophagosome-like vacuoles characterized by multilamellar membranes after 24 h of infection and that *Brucella* replication was increased when macroautophagy was activated by serum starvation, suggesting that *B. abortus* transits through the autophagic pathway before reaching its replicative compartment [11,13]. Since then, many proteins implicated in the regulation of macroautophagy (Atg proteins) have been discovered [14,15]. The initiation of autophagosome formation requires the ULK complex and the class III phosphatidylinositol 3-P kinase (PI3K) complex. The nucleation of the isolation membrane requires the recruitment of additional Atg proteins and autophagy-specific PtdIns(3)P effectors [14,15]. The expansion of the isolation membrane relies on two ubiquitylation-like reactions. The first one drives the conjugation of Atg12 to Atg5 in the presence of Atg7 and Atg10. Atg5-Atg12 conjugates are non-covalently associated to Atg16L (Atg16-like) forming multimeric complexes of approximately 800 kDa [16–18]. The second reaction conjugates the cytosolic soluble LC3-I (microtubule-associated protein 1 light chain 3) to a phosphatidylethanolamine (PE) in the presence of Atg4, Atg3 and Atg7 producing the membrane-associated LC3-II form [19–21]. The Atg5-Atg12 conjugates are essential for the maturation of the isolation membrane into autophagosome and targeting of LC3 to the membrane [18].

Recently, using epithelial cells and macrophages deficient in one of the regulatory proteins of the conventional macroautophagic pathway, Starr et al. [12] have found that core proteins of this canonical macroautophagy machinery such as ULK-1, Beclin1, Atg5, Atg7, LC3B were not necessary for the intracellular trafficking of *B. abortus* between the endocytic compartments and the ER-derived vesicles and for its replication [12]. Nevertheless, the conversion of rBCV to aBCV at a later stage of infection, i.e. 48 h and 72 h p.i., seems to be dependent on ULK-1, Beclin1, Atg14L and hVps34 but independent on Atg5, Atg7, Atg16L1 and Atg4B [12]. On the other hand, Guo et al. [22] have observed that infection by *B. melitensis* induced macroautophagy that in turn favoured its replication in RAW264.7 macrophages [22]. This later study raises the possibility that in contrast to *B. abortus*, *B. melitensis* could subvert macroautophagy to replicate in host cells. In our present work, we addressed this issue using embryonic fibroblasts from wild-type and Atg5-knockout mice infected or not with *B. abortus* and *B. melitensis*.

## Results

### Relative abundance of LC3-I and LC3-II in infected mouse embryonic fibroblasts

**Figure 1:**
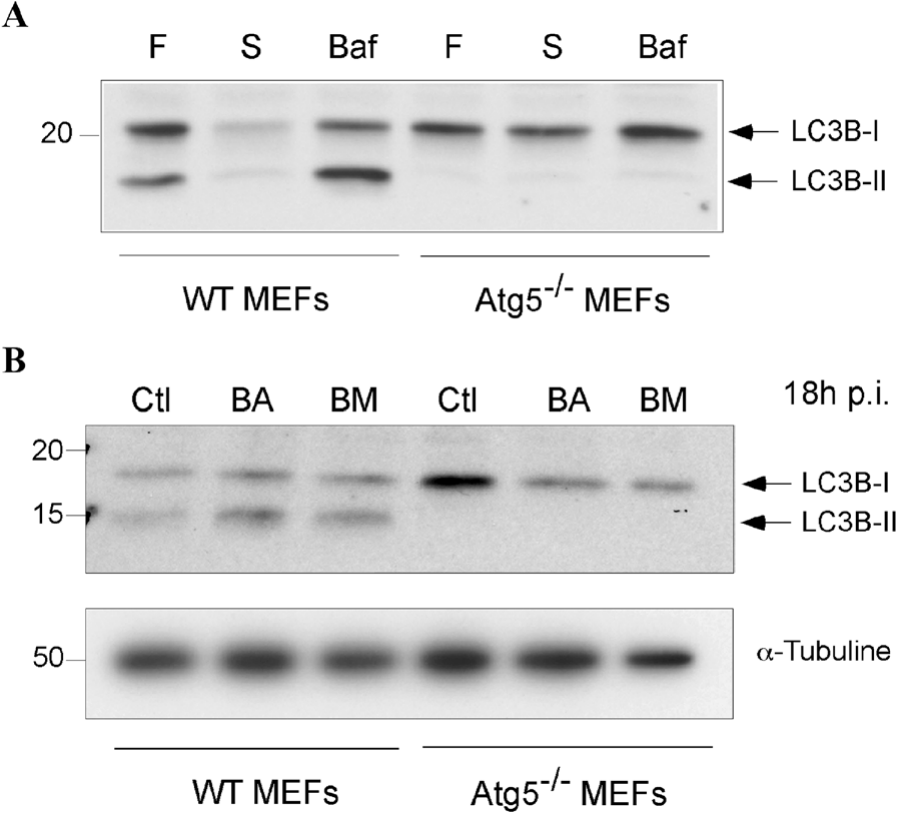
**Relative abundance of LC3B-I and LC3B-II in WT MEFs and in Atg5** ^**−/−**^ **MEFs as determined by immunoblotting. A**. Cells were maintained in DMEM/FCS (F), starved for 2 h in EBSS (S) or incubated for 5 h in the presence of 100 nM bafilomycin (Baf). **B**. Cells were infected with *B. abortus* (BA) or with *B. melitensis* (BM) for 18 h or left non infected (Ctl).

As it has been shown that *B. melitensis* stimulated macroautophagy in macrophages to favour its replication [22], we sought to determine whether this also occurred in infected MEFs. First, we established clones stably transfected with GFP-LC3 to monitor the formation of autophagic vacuoles by fluorescence microscopy. As expected [19], in basal conditions, the fluorescent staining in GFP-LC3 expressing cells was faint and diffuse while under starvation conditions, it was more punctuate, due to the recruitment of LC3 onto autophagosomal membranes (Additional file 1). In contrast, when the same cells were infected with *B. abortus* or with *B. melitensis*, the GFP-LC3 staining remained diffuse and colocalisation between GFP-LC3 and Texas Red-labelled bacteria was only very occasionally detected. Then, we examined the relative abundance of LC3-I and LC3-II by Western blotting. Preliminary experiments showed that in WT MEFs, LC3-II was detected even in basal conditions (Figure 1A). After 2 h of starvation in EBSS, the abundance of both LC3-I and LC3-II decreased, probably due to an acceleration of the autophagic flow since LC3-II is degraded when autophagosomes fuse with lysosomes. In contrast, the LC3-II/LC3-I ratio increased in the presence of bafilomycin, a vacuolar H^+^-ATPase inhibitor known to block autophagosome/lysosome fusion. As expected, in Atg5^−/−^ MEFs, LC3-II was never detected whatever the cell culture conditions because the presence of Atg5 is absolutely required for the LC3 recruitment onto autophagosome membrane [19]. In WT MEFs infected with *B. abortus* or with *B. melitensis*, the relative abundance of LC3-I and LC3-II at 18 h p.i. did not change when compared to non-infected MEFs (Figure 1B).

### Replication of *B. abortus*- and *B. melitensis*-mCherry in Atg5^−/−^ fibroblasts

**Figure 2:**
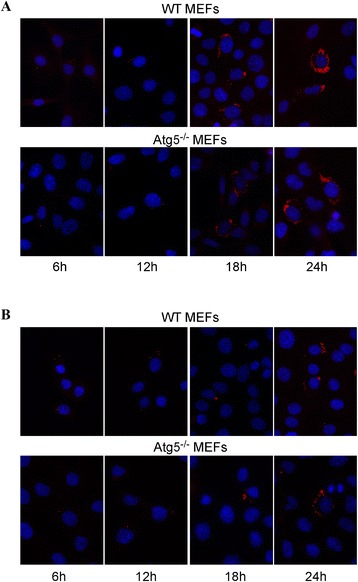
**Fluorescence microscopy analysis of WT MEFs and Atg5** ^**−/−**^ **MEFs infected with** ***B. abortus*** **-mCherry (A) or with** ***B. melitensis-*** **mCherry (B).** MEFs were infected for 1 h with *Brucella*-mCherry at an MOI of 300 and observed at 6 h, 12 h, 18 h and 24 h p.i. The nuclei were stained with DAPI.

**Figure 3:**
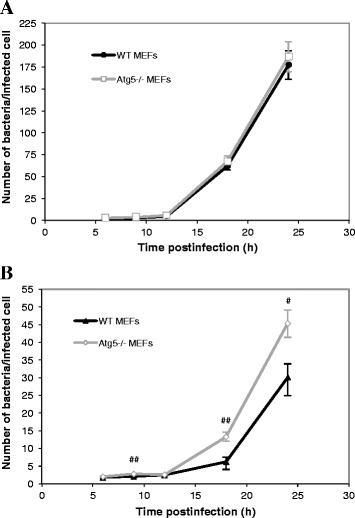
**Quantification of the infection of WT MEFs and Atg5** ^**−/−**^ **MEFs with** ***B. abortus*** **-mCherry (A) or with** ***B. melitensis-*** **mCherry (B).** MEFs were infected for 1 h with *Brucella*-mCherry at an MOI of 300. Cells were observed by fluorescence microscopy at 6 h, 9 h, 12 h, 18 h and 24 h p.i. Values represent the number of bacteria per infected cell as means ± SEM with n ≥ 50, where n is the number of observed infected cells. Statistical significance was calculated using the Mann–Whitney Rank Sum Test. # and ## indicate a significant difference with *p* <0.05 and *p* <0.01, respectively.

We studied the contribution of the macroautophagic pathway on the replication of *Brucellae* using Atg5-deficient MEFs. First, we infected cells with *B. abortus*-mCherry (Figure 2A) or with *B. melitensis*-mCherry (Figure 2B) for 1 h at a multiplicity of infection (MOI) of 300. After inoculation, the medium was removed and replaced by a medium containing gentamicin to kill extracellular bacteria. As it can be seen on micrographs taken after increasing times postinfection, *B. abortus*-mCherry is able to enter, survive and replicate in MEFs, even in Atg5-deficient MEFs. In both cell lines, at 6 h p.i, there are only a few bacteria per infected cell but this number massively increases between 12 and 18 h p.i. and at 24 h p.i., the bacteria are so abundant that it is difficult to enumerate them. *B. melitensis*-mCherry is also able to replicate in both WT MEFs and Atg5^−/−^ MEFs. However, it is clear that the number of bacteria per infected cell at 24 h p.i. is lower compared to *B. abortus*-mCherry. Statistical analysis of these observations revealed that there is no significant difference in the number of *B. abortus*-mCherry per infected cell between the Atg5-deficient MEFs and the WT MEFs whatever the time postinfection (Figure 3A). In contrast, the number of *B. melitensis*-mCherry per infected cell significantly increased in Atg5^−/−^ MEFs when compared to WT MEFs at 9 h, 18 h and 24 h p.i. (Figure 3B). These data demonstrate that both *Brucella* strains can survive and replicate when the conventional Atg5-dependent macroautophagic pathway is impaired. Atg5-deficient cells seem to be even more permissive for *B. melitensis* replication than WT MEFs.

### Counting of viable bacteria in Atg5^−/−^ fibroblasts

**Figure 4:**
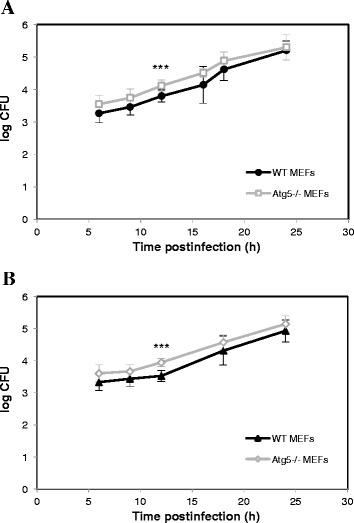
**Intracellular growth of** ***Brucella*** **in WT and Atg5** ^**−/−**^ **MEFs.** MEFs were infected for 1 h with *B. abortus* S2308 **(A)** or with *B. melitensis* 16M **(B)** at an MOI of 300. Log CFUs were obtained from cell lysates of infected WT MEFs and Atg5^−/−^ MEFs at the indicated time after infection. Results represent means ± SD measured from at least three independent experiments made in triplicates. Statistical significance was calculated using the Holm-Sidak multiple comparisons test following a two-way ANOVA. *p* < 0.001 for both *B. abortus* and *B. melitensis*. *** indicates a highly significant difference using a Student’s *t-*test.

The counting of CFUs in the gentamicin survival assay represents a common way to investigate the survival and the replication of bacteria in host cells. In agreement with our morphological observations, we noticed that *B. abortus* grew at an exponential rate as a function of time postinfection both in WT and Atg5^−/−^ MEFs (Figure 4A). There was even a slight increase in the log CFU in Atg5^−/−^ MEFs as compared to WT MEFs. A Student’s t-test on each time point indicated that the difference between the WT and Atg5^−/−^ MEFs was significant only at 12 h p.i. Nevertheless, a two-way ANOVA statistical analysis on all time points combined revealed that there was a highly significant increase in the log CFU in Atg5^−/−^ MEFs when compared to WT MEFs (*p* < 0.001). The same observation was made with *B. melitensis* (Figure 4B). This global increase could result from a more efficient uptake of bacteria rather than from a higher replication rate in Atg5^−/−^ MEFs compared to WT MEFs. Alternatively, this increase in log CFU could be linked to a lower bactericidal capacity of Atg5-deficient cells compared to WT cells at early stages of infection.

### Intracellular replication of *B. abortus* and *B. melitensis* in the presence of 3-methyladenine

**Figure 5:**
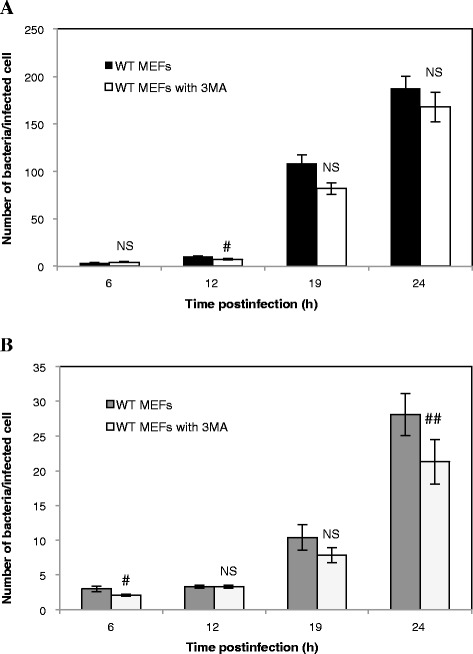
**Impact of 3MA on the infection of WT MEFs with** ***B. abortus*** **-mCherry (A) or with** ***B. melitensis-*** **mCherry (B).** The number of bacteria per infected cell was measured on at least 57 infected cells coming from two independent experiments. Values represent means ± SEM. Statistical significance was calculated using the Mann–Whitney Rank Sum Test. # and ## indicate a significant difference with *p* <0.05 and *p* <0.01, respectively. NS stands for non significant difference.

**Figure 6:**
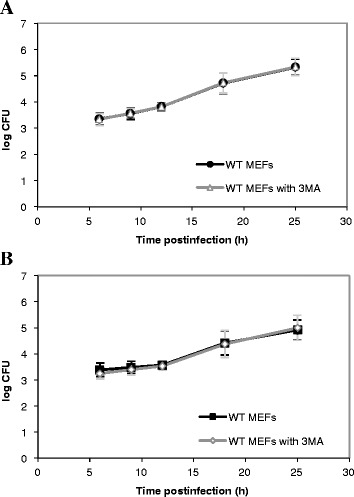
**Impact of 3MA on the infection of WT MEFs with** ***B. abortus*** **S2308 (A) or with** ***B. melitensis*** **16M (B).** Results represent log CFUs (means ± SD) measured at various times postinfection in at least three independent experiments made in triplicates.

Previous studies have shown that incubation of cells in the presence of 3-methyladenine (3MA), an inhibitor of class III PI3K often used to block macroautophagy [23], impaired the replication of *B. abortus* [13] and *B. melitensis* [22] in HeLa cells and in RAW264.7 macrophages, respectively. These data are in contradiction with our results showing that both bacterial strains are able to replicate in Atg5-deficient MEFs. Therefore, we sought to determine the putative impact of 3MA on the replication of *Brucellae* in WT MEFs. First, we assessed the number of *B. abortus*-mCherry per infected cell in WT MEFs preincubated for 2 h in the presence or absence of 10 mM 3MA. As shown in Figure 5A, this treatment had no significant impact on the number of bacteria per infected WT MEF. Similar results were obtained with WT MEFs infected with *B. melitensis*-mCherry (Figure 5B). However, in this case, we observed a significant decrease (*p* < 0.01) in the number of bacteria per infected cell but only at 24 h p.i. Next, we examined the impact of a pre-treatment with 3MA on *Brucella* replication in host cells using the gentamicin survival assay. Our results show that a pre-incubation of WT MEFs with 3MA does not impair the replication of both *B. abortus* and *B. melitensis* (Figure 6 A-B).

## Discussion

After internalisation, *B. abortus* is found inside individual vacuoles that interact transiently with endosomes and perhaps lysosomes [6]. Then, *Brucella* evades the endocytic pathway and reaches its replicative niche, an ER-derived compartment, by a still unknown mechanism. It is also unclear whether *Brucella* transits through the autophagic pathway before its replication. Based on the appearance of *B. abortus* in multilamellar structures looking like autophagosomes and on the decrease of its replication rate after autophagy inhibition with 3MA, Pizarro-Cerda et al. [11] proposed that this bacterium passed through the autophagy pathway before reaching its niche of replication [13]. In agreement with this assumption, Guo et al. (2012) noticed that inoculation of macrophages with *B. melitensis* stimulated autophagy and that a pre-treatment with 3MA reduced its growth rate [22]. In contrast, using macrophages derived from KO mice or HeLa cells incubated in the presence of siRNA targeting the autophagic machinery, Starr et al. [12] showed that *B. abortus* does not use the conventional macroautophagic pathway either for its intracellular trafficking between the endocytic compartments and the ER derived-vesicles or for its replication [12]. In our study, we sought to compare the fate of *B. abortus* and *B. melitensis* in Atg5-deficient MEFs, i.e. in cells that are unable to set up the conventional pathway of macroautophagy even under starvation conditions.

Our results show that both *Brucella* strains are able to invade and replicate in Atg5^−/−^ MEFs, indicating that Atg5 is dispensable for the intracellular survival and replication not only of *B. abortus* but also of *B. melitensis*. We observed even a slight but significant increase in the log CFU in Atg5^−/−^ MEFs infected with *B. abortus* or with *B. melitensis* when compared to WT MEFs, all time points combined. The counting of fluorescent bacteria per infected cell, which takes into account living bacteria but also dead bacteria and bacteria that are no longer able to replicate, indicates that for *B. abortus*, there is no difference between the two cell lines even at short times postinfection (Figure 3A) whereas for *B. melitensis*, there is a significant increase in the Atg5^−/−^ MEFs at 9, 18 h and 24 h. p.i., as compared to WT MEFs (Figure 3B). Therefore, for *B. abortus*, the higher CFUs in Atg5^−/−^ MEFs *vs* WT MEFs could be explained by an increase in the percentage of infected cells among the cell population or by a higher survival rate during the early times after infection rather than by a higher replication rate. In contrast, for *B. melitensis*, the increase in the log CFU in Atg5-deficient cells could also result from a slight increase in the replication rate.

Next, our data reveal that there is no conversion of LC3-I to LC3-II in WT MEFs upon *Brucella* invasion and that neither *B. abortus* nor *B. melitensis* is detected in autophagic compartments stained with GFP-LC3, even under starvation conditions. This is consistent with the results of Starr et al. [12], which also showed that the siRNA-mediated silencing of LC3B in HeLa cells did not impair the maturation of the BCV into a replicative niche in cells infected with *B. abortus*. In contrast, Guo et al. [22] proposed that *B. melitensis* infection induced autophagy because they observed an accumulation of GFP-LC3-positive autophagic vacuoles and a conversion of LC3-I to LC3-II in infected RAW264.7 macrophages, compared to control cells. Moreover, these authors showed that a treatment with the autophagy inhibitor 3MA attenuated the replication efficiency of *B. melitensis*. It is not clearly indicated how long they incubated cells with this compound but it has been demonstrated that under nutrient-rich conditions, a prolonged treatment (up to 9 h) with 3MA could promote rather than inhibit the autophagy flux [24]. In contrast to Guo et al., [22], we did not observe a significant decrease in the CFU and in the number of *Brucella* per infected cells (except for *B. melitensis* at 24 h p.i.) in WT MEFs pretreated with 3MA. This discrepancy could be explained either by the incubation conditions or by a cell-type specificity. The subversion of the autophagic pathway by *B. melitensis* could occur in RAW264.7 macrophages but not in MEFs.

Given the multifactorial effects of 3MA on cell metabolism [25], cells derived from Atg5 KO mice represent a more reliable tool to study the role of autophagy in different biological situations [18]. Based on our results with Atg5^−/−^ MEFs, it is obvious that *B. melitensis* 16M as well as *B. abortus* are able to replicate in cells deficient in the canonical macroautophagy pathway. However, we cannot rule out the involvement of autophagosomes formed by an Atg5 and Atg7-independent alternative macroautophagy. Indeed, it has been demonstrated that the incubation of Atg5^−/−^ MEF with etoposide, a proapoptotic molecule, induced autophagosome formation without conversion of LC3-I to LC3-II [26]. Likewise, Starr et al. [12] have shown that the conversion of rBCVs into aBCV that occurs at a very late stage after infection with *B. abortus* does not require several core autophagic proteins, of which Atg5 and LC3B [12]. These findings demonstrate that autophagic vacuoles can be formed in Atg5-deficient cells. However, these alternative macroautophagy pathways, independent of Atg5 and LC3, are inhibited by 3MA [12,26]. Thus, if *Brucella* subverts an alternative macroautophagy pathway to reach its replicative niche in mouse embryonic fibroblasts, it should proceed by another mechanism because in our conditions of incubation, the replication efficiency is not impaired in WT MEFs treated with 3MA.

Finally, it has been demonstrated that the intracellular trafficking of *B. abortus* and *B. melitensis* could be different in some human trophoblastic cell lines [27]. Therefore, it could be interesting to study the involvement of the conventional and the alternative macroautophagy pathways in other cell types, such as trophoblasts and peritoneal or bone marrow-derived macrophages.

## Conclusion

Collectively, our data indicate on one hand that cell invasion with *B. abortus* and *B. melitensis* does not induce macroautophagy in WT MEFs and on the other hand, that both *Brucella* strains can replicate in Atg5-deficient MEFs.

## Methods

### Bacteria strains

*Brucella abortus* S2308 and *Brucella melitensis* 16M are CO_2_-independent virulent smooth strains. *Brucella*-mCherry strains constitutively express the fluorescent mCherry protein due to the intregration of a plasmid containing the coding sequence of mCherry and a kanamycin resistance marker [28]. Before each infection, bacteria stored at −80°C were plated onto 2YT Agar (1.6% bacto-peptone, 1% yeast extract, 0.5% NaCl and 1.3% Agar) Petri dishes. For *Brucella-*mCherry, kanamycin (10 μg/mL) was added in this culture medium to maintain selection. After approximately 72 hours of incubation at 37°C, a dozen or so isolated colonies were taken and cultured overnight at 37°C under agitation in 5 mL of 2YT liquid medium (1% tryptone, 0.6% bacto-peptone, 1% yeast extract and 0.5% NaCl) without antibiotics.

### Host cells

We used mouse embryonic fibroblasts from wild type (WT MEFs) and from Atg5 knockout mice (Atg5^−/−^ MEFs) [29] available at the Riken BRC Cell Bank. Cells were cultured in Dulbecco’s modified Eagle medium (DMEM, Lonza) supplemented with 10% vol/vol fetal calf serum (FCS, Sigma). After counting in a Burker chamber, MEFs were seeded at a density of 50,000 cells/well in 12-well plates containing coverslips for the microscopy experiments and in 24-well plates in triplicates for the counting of CFUs. After seeding, cells were incubated overnight at 37°C under 5% of CO_2_ before bacteria inoculation. When indicated, 10 mM 3-methyladenine (Sigma, directly prepared in DMEM medium) was added to the cell monolayers to inhibit autophagy prior to infection. To investigate the presence of *Brucella* in LC3B-positive autophagosomes, we established stable clones of MEFs expressing GFP-LC3 WT (plasmid pEX-GFP-hLC3WT, Addgene). Starvation-induced autophagy was obtained by a 2 h-incubation in EBSS medium (Earle’s Balanced Salt solution) after three washes with PBS to remove serum.

### Cell infection with *Brucella*

Growth of bacteria was assessed by measuring the optical density (OD) at a wavelength of 600 nm considering that an OD = 1 corresponds to 1×10^9^ bacteria/mL. Then, bacteria were sedimented by centrifugation at 900 *g* for 10 min to discard 2YT medium and resuspended in the same volume of DMEM + 10% FCS. After dilution of the bacterial suspension in an appropriate volume of DMEM + FCS to get an MOI (multiplicity of infection) of 300, the culture medium present in 12-well plates containing MEFs was withdrawn and replaced by the bacterial suspension. The Petri dishes were centrifuged for 10 min at 400 *g* at 4°C to favour the adhesion of bacteria to the cell surface and then placed in a 5% CO_2_ incubator at 37°C (this is the time zero postinfection). The passage from 4°C to 37°C aims at synchronizing the entry of bacteria into the cells. After one hour of infection, wells were washed thrice with sterile phosphate-buffered saline (PBS, 136.9 mM NaCl, 2.7 mM KCl, 10.1 mM Na_2_HPO_4_ and 1.8 mM KH_2_PO_4_) and further incubated for one hour with DMEM + FCS containing 50 μg of gentamicin per mL to kill extracellular bacteria. Afterwards, the medium was changed and replaced by the medium containing 10 μg of gentamicin per mL until the end of the postinfection period [28].

For the counting of viable intracellular bacteria using colony forming units (CFUs), after infection with *Brucella*, cells were washed thrice with PBS then lysed for 10 min at room temperature in 800 μl of PBS containing 0.1% Triton X-100 under manual agitation. Lysates were diluted from 10 to 1,000 times in PBS and plated on Petri dishes containing 2YT Agar. Petri dishes were incubated for three to four days at 37°C before the counting of colony forming units.

### Fluorescence microscopy

To count the number of *Brucella* per infected cell, we infected MEFs with *Brucella-*mCherry. At various time points p.i., cells were washed twice with filtered dPBS (PBS supplemented with 0.88 mM CaCl_2_ and 0.5 mM MgCl_2_), fixed for 20 min at room temperature in 4% paraformaldehyde in cold PBS, then washed thrice with dPBS. Nuclei were stained with 4’-6-diamidino-2-phenylindole (DAPI) prepared in PBS containing 0.1% Triton X-100 and washed three times with PBS. Coverslips were mounted in Mowiol on glass plates. Fluorescence was observed using a Nikon i80 fluorescence microscope. In an attempt to detect *Brucella* in compartments stained with LC3, we infected cells expressing GFP-LC3 with *B. abortus* S2308 or with *B. melitensis* 16M that do not express mCherry. After fixation, membrane permeabilisation with Triton X-100 (0.1% in dPBS) and blocking of unspecific sites with bovine serum albumine (2% in dPBS), bacteria were detected with a monoclonal antibody raised against the lipopolysaccharides of *Brucella* (A76-12G12) [30] and a goat anti-mouse Texas Red-conjugated secondary antibody. Fluorescence was observed using a Leica TCD confocal fluorescence microscope.

### Western blotting

MEFs were washed three times with PBS and then incubated for 10 min in cold lysis buffer (10 mM Tris–HCl pH 7.4, 150 mM NaCl, 0.5% Triton X-100 and a protease-inhibitor cocktail (Roche)). After 10 min of rotation on a wheel, cell lysates were centrifuged for 15 min at 13,000 RPM at 4°C to sediment cell debris. Protein concentration of these clear lysates was determined using the BCA (Bicinchoninic acid) protein assay (Pierce). Fifteen micrograms of proteins were separated by SDS-PAGE 12% and then, transferred onto polyvinyl difluoride (PVDF) membranes. Membranes were blocked for 1 h in PBS containing 0.1% Tween 20 and 2% of blocking agent (GE Healthcare), then incubated for 2 h with a primary monoclonal anti-LC3B antibody (NanoTools, Germany) and a secondary anti-mouse antibody conjugated to horseradish peroxidase (HRP). The activity of HRP was revealed by enhanced chemiluminescence (Perkin-Elmer).

### Statistical analysis

Error bars indicate standard deviation (SD) or standard error of the mean (SEM) as indicated in the legend. Statistical significance was determined using SigmaPlot 11 software. Whenever possible, we have performed unpaired Student’s *t*-tests. When the normality test (Shapiro-Wilk) or the equal variance test failed, we carried out a Mann–Whitney rank sum test. A two-way ANOVA followed by a pairwise multiple comparison procedure (Holm-Sidak method) was also carried out. Statistical significant differences were accepted for *p* < 0.05.

### Ethics statement

No live animal was used in this work.

## Additional file

Additional file 1:
**GFP-LC3 labelling in WT MEFs infected or not with** ***B. abortus*** **or** ***B. melitensis.*** WT MEFs stably expressing GFP-LC3 were maintained under normal conditions (left) or under starved conditions (right). NI, BA and BM correspond to non infected cells, cells infected with *B*. abortus and cells infected with *B*. melitensis, respectively. MEFs were fixed at 10 h p.i. Bacteria were detected with a monoclonal anti-LPS antibody and an anti-mouse IgG Texas Red-conjugated secondary antibody. Nuclei were stained with DAPI. Cells were observed by confocal fluorescence microscopy.

## Abbreviations

MEFs: Mouse embryonic fibroblasts
MOI: Multiplicity of infection
CFU: Colony forming units
WT: Wild-type
3MA: 3-methyladenine
