# Supplementary material for: Replication of Brucella abortus and Brucella melitensis in fibroblasts does not require Atg5-dependent macroautophagy
Source: BMC Microbiol. 2014 Sep 2;14:223. doi: 10.1186/s12866-014-0223-5 (PMC4159544; doi:10.1186/s12866-014-0223-5)
Supplement: Additional file 1: — GFP-LC3 labelling in WT MEFs infected or not with B. abortus or B. melitensis. WT MEFs stably expressing GFP-LC3 were maintained under normal conditions (left) or under starved conditions (right). NI, BA and BM correspond to non infected cells, cells infected with B. abortus and cells infected with B. melitensis, respectively. MEFs were fixed at 10 h p.i. Bacteria were detected with a monoclonal anti-LPS antibody and an anti-mouse IgG Texas Red-conjugated secondary antibody. Nuclei were stained with DAPI. Cells were observed by confocal fluorescence microscopy. [file 12866_2014_223_MOESM1_ESM.pdf]

Additional file

Figure S1

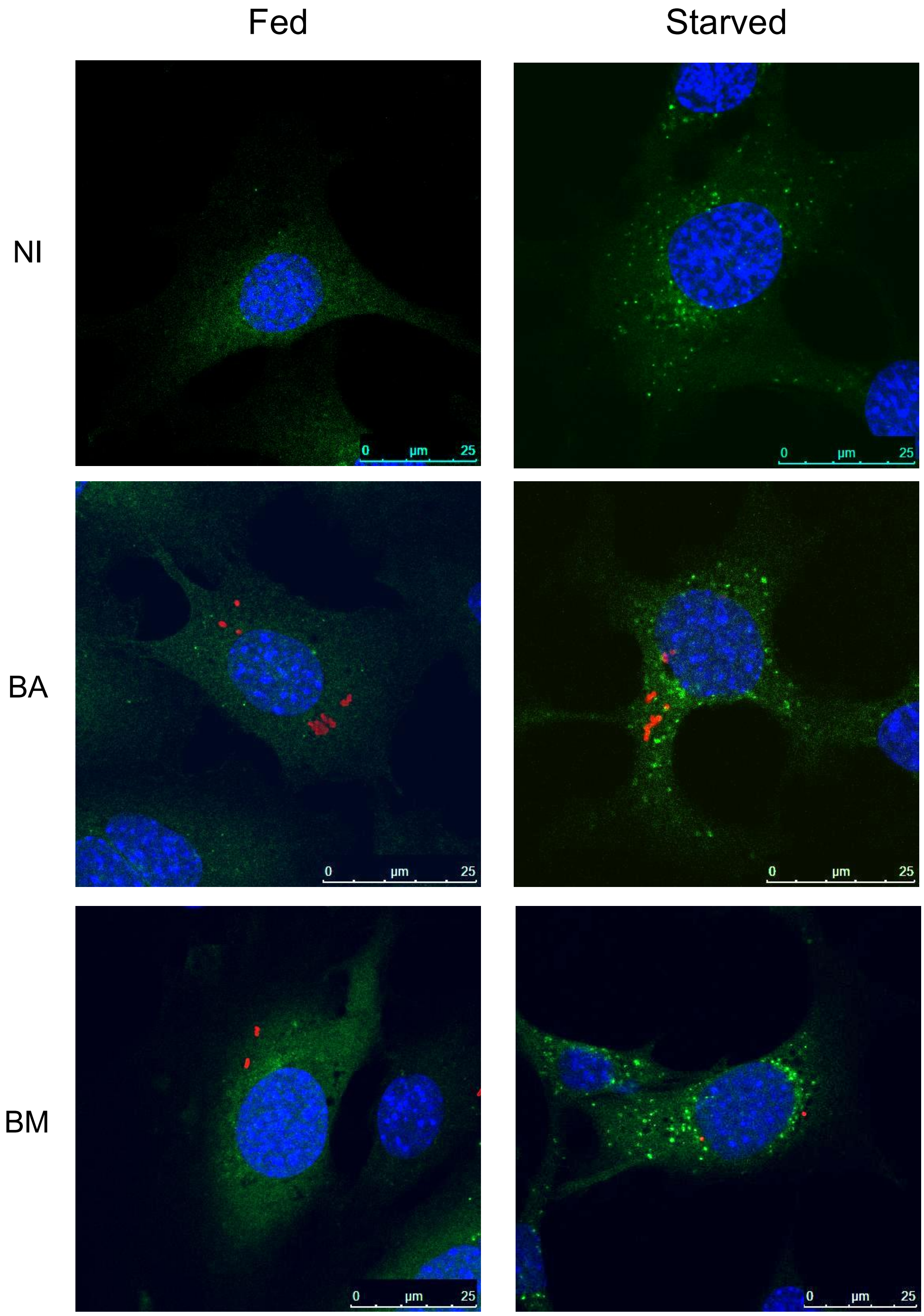

Figure S1. GFP-LC3 labelling in WT MEFs infected or not with *B. abortus* or *B. melitensis*. WT MEFs stably expressing GFP-LC3 were maintained under normal conditions (left) or under starved conditions (right). NI, BA and BM correspond to non infected cells, cells infected with *B. abortus* and cells infected with *B. melitensis*, respectively. MEFs were fixed at 10 h p.i. Bacteria were detected with a monoclonal anti-LPS antibody and an anti-mouse IgG Texas Red-conjugated secondary antibody. Nuclei were stained with DAPI.
